# Supplementary material for: Trajectory of Humoral Responses to Two Doses of ChAdOx1 nCoV-19 Vaccination in Patients Receiving Maintenance Hemodialysis
Source: Microbiol Spectr. 2023 Feb 21;11(2):e03445-22. doi: 10.1128/spectrum.03445-22 (PMC10100369; doi:10.1128/spectrum.03445-22)

Supplementary Table 1. Difference of log_{10} transformed anti-SARS-CoV2 receptor binding domain (RBD) antibody level between the high- or low-response group at different time points after the one or two-dose of ChAdOx1 nCoV-19 vaccination.

Time	High-response group	Low-response group	P value
V1M1	1.45 ± 0.44	-0.02 ± 0.45	< 0.0001
V1M2	1.90 ± 0.43	0.67 ± 0.78	< 0.0001
V2M1	2.89 ± 0.37	2.18 ±0.83	< 0.0001
V2M5	2.34 ± 2.22	1.52 ± 0.83	< 0.0001

Abbreviation: V1M1: one month after the first dose of vaccination; V1M2: two months after the first dose of vaccination; V2M1: one month after the second dose of vaccination; V2M5: five months after the second dose of vaccination.

Supplementary Figure 1. Flowchart of the establishment of the study cohort.

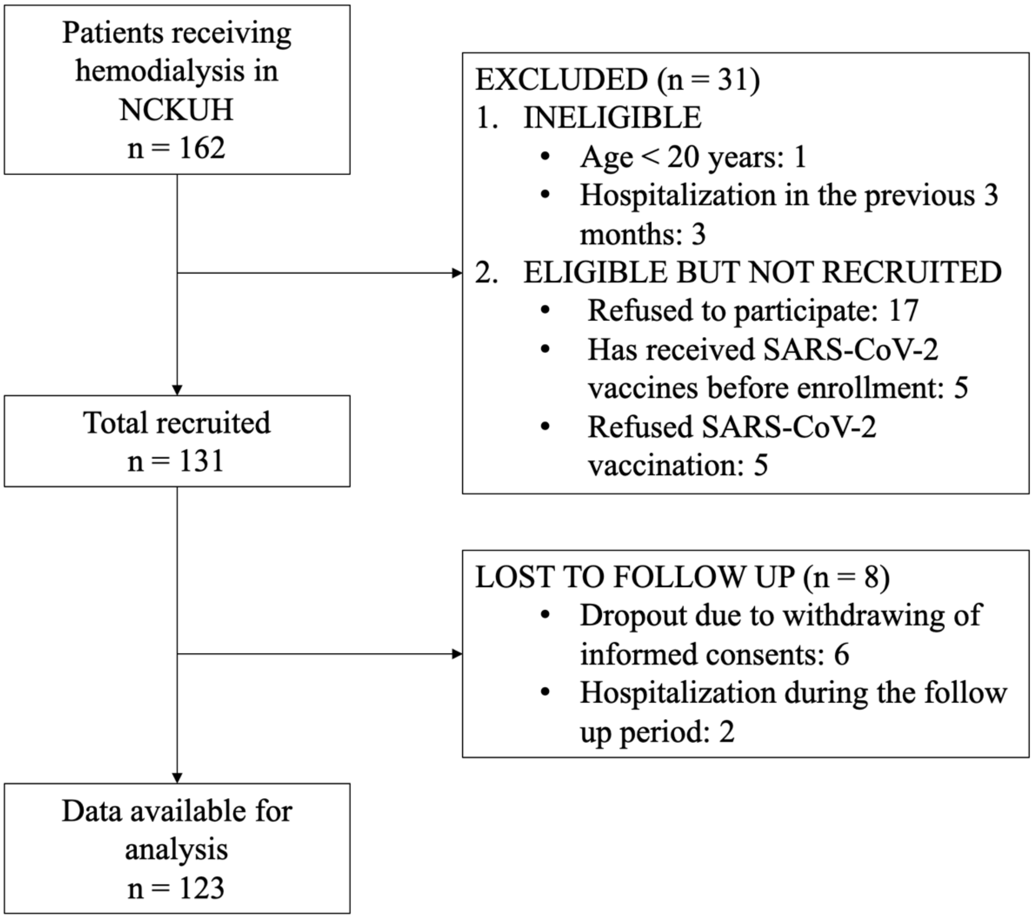

Supplementary Figure 2. Patient numbers with adverse events following the first and second dose of AZD 1222 vaccination.

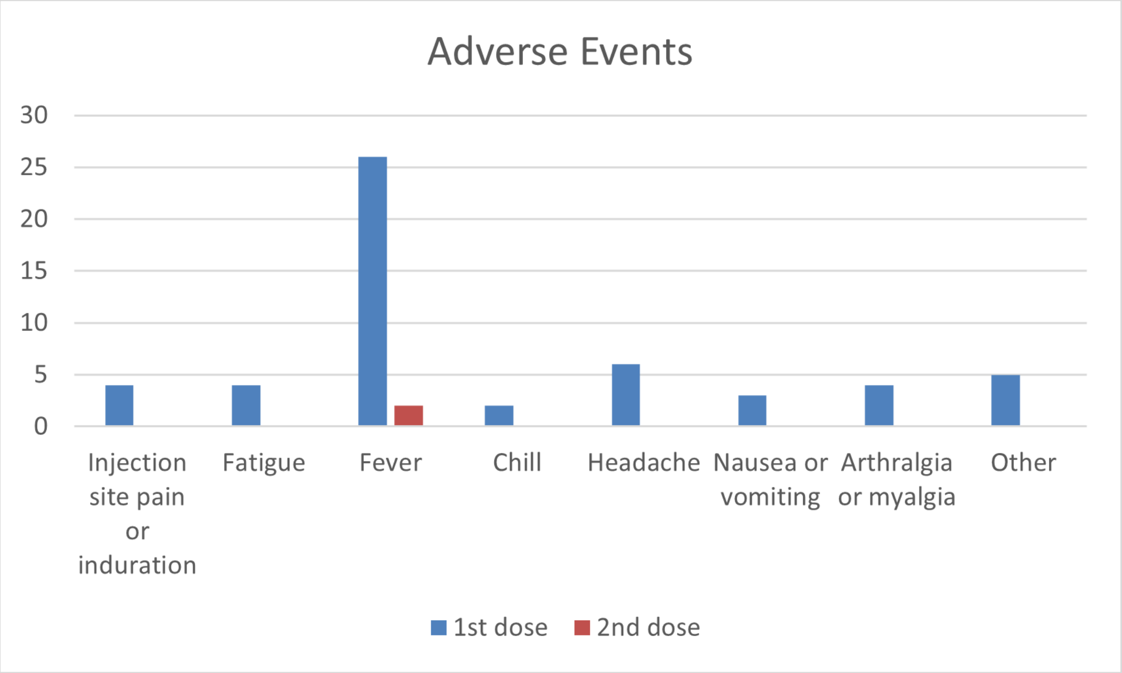

Supplementary Figure 3. Distribution of different neutralization levels measured by the surrogate viral neutralization test (sVNT), cPass kit, against the ancestral virus, delta, and omicron variants in the study cohort. Negative, positive, high-positive, and very high-positive level of sVNT is defined as < 30%, ≥ 30% and < 68%, ≥ 68% and < 80%, and ≥ 80%, respectively.

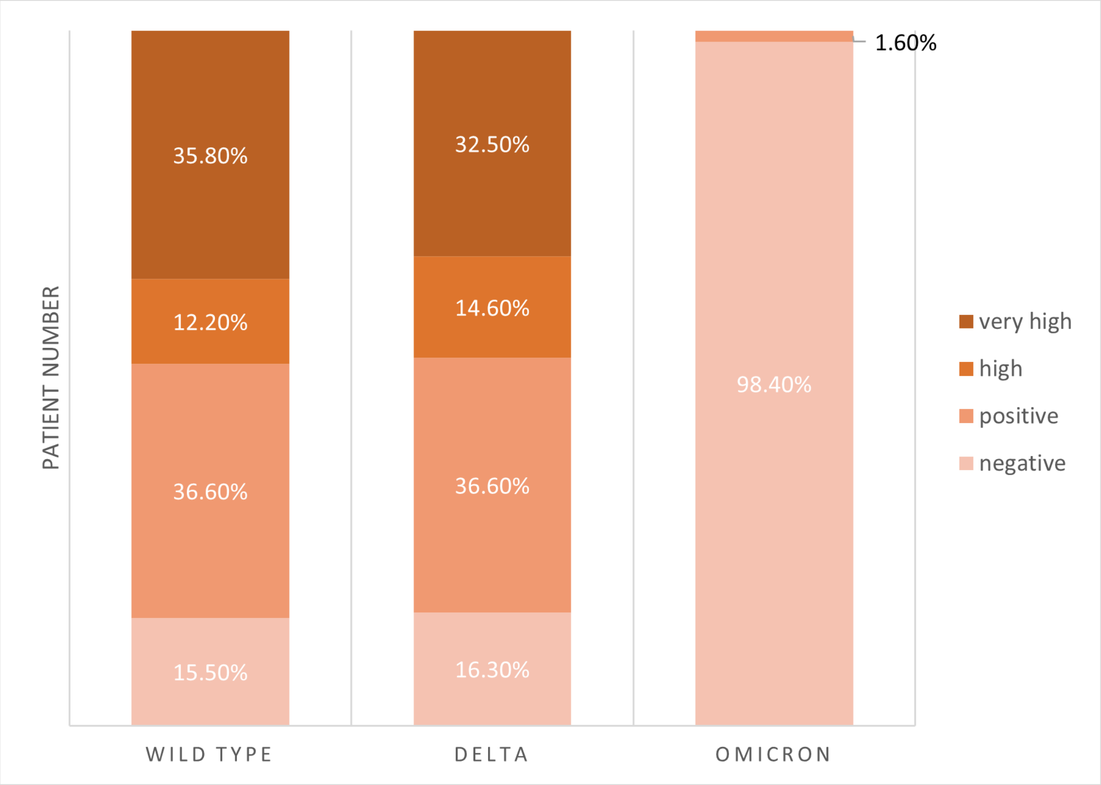

Supplementary Figure 4. Comparison of anti-SARS-CoV2 receptor binding domain (RBD) antibody level between healthcare workers and hemodialysis patients at 20-24 weeks or 5 months, respectively, after the second dose of ChAdOx1 nCoV-19 vaccine.

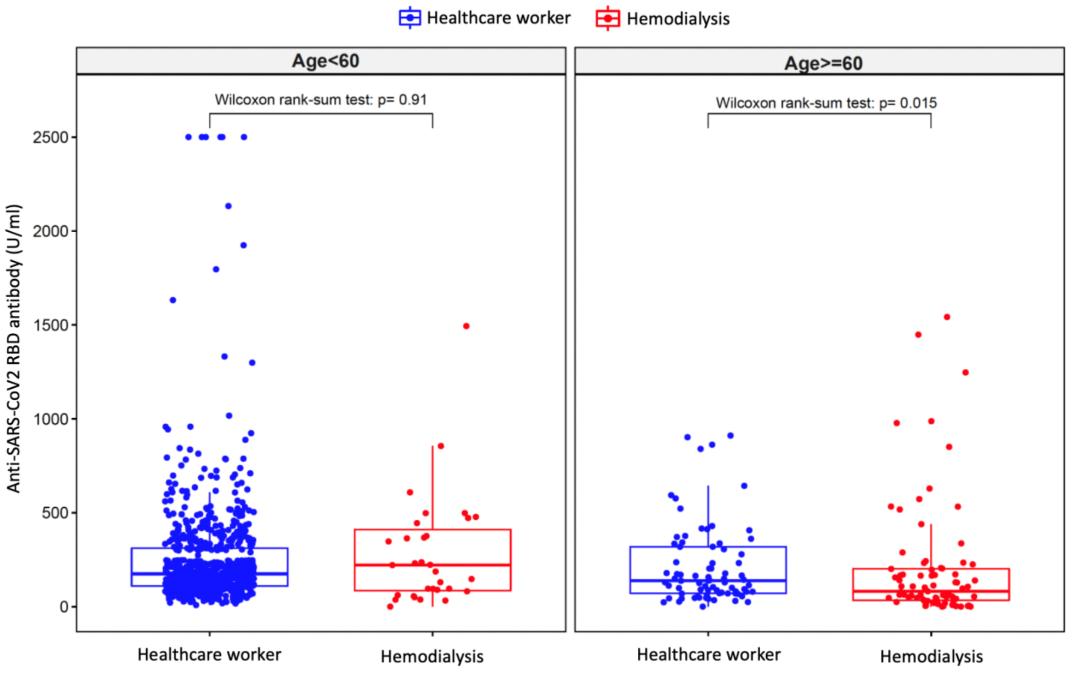

Supplementary Figure 5. Trajectories of anti-SARS-CoV2 receptor binding domain (RBD) antibody level of the low and high response group, stratified by median level at one month after the first dose of ChAdOx1 nCoV-19 vaccination.

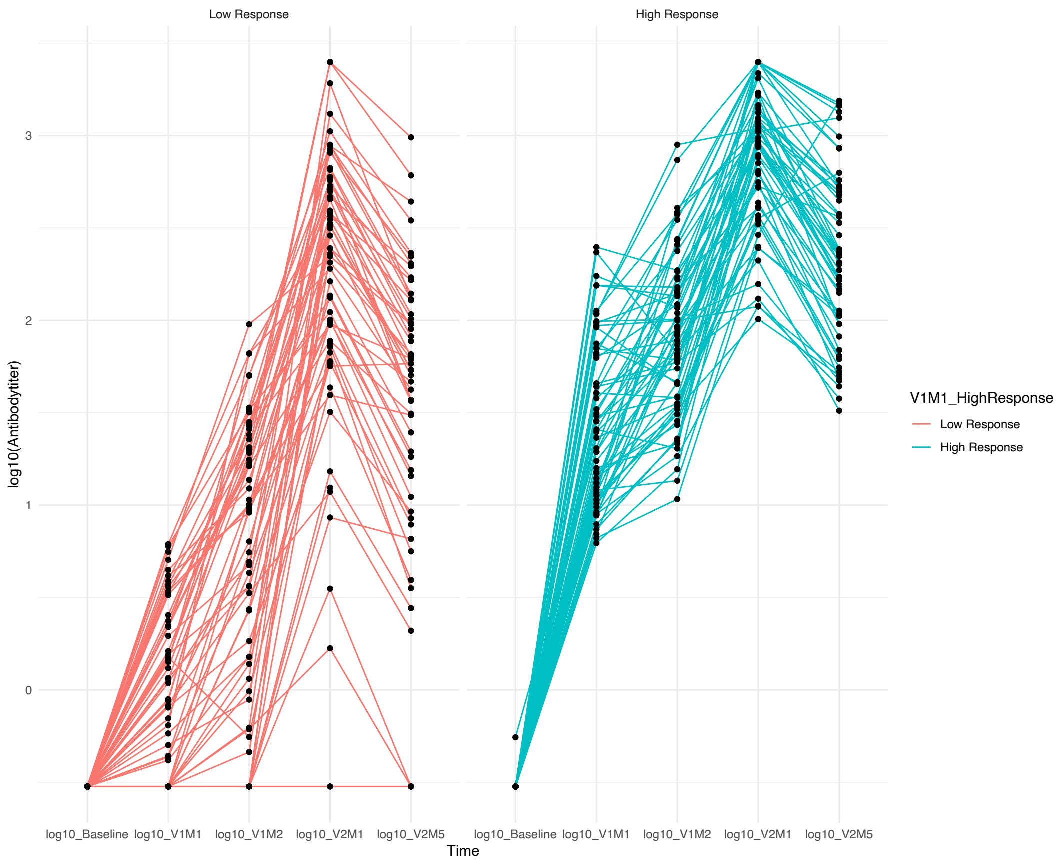

Supplement: Supplemental file 1 — Table S1 and Fig. S1 to S5. Download spectrum.03445-22-s0001.pdf, PDF file, 0.9 MB [file spectrum.03445-22-s0001.pdf]
